# “I don't have Huntington's disease”: the boundaries between acceptance and understanding

**DOI:** 10.1055/s-0043-1768158

**Published:** 2023-07-26

**Authors:** Gustavo Leite Franklin, Hélio A. Ghizoni Teive, Francisco Eduardo Cardoso

**Affiliations:** 1 Pontifícia Universidade Católica do Paraná, Departamento de Medicina Interna, Curitiba PR, Brazil.; 2 Universidade Federal do Paraná, Departamento de Medicina Interna, Serviço de Neurologia, Unidade de Distúrbios do Movimento, Curitiba PR, Brazil.; 3 Universidade Federal de Minas Gerais, Departamento de Medicina Interna, Serviço de Neurologia, Belo Horizonte MG, Brazil.

**Keywords:** Huntington Disease, Agnosia, Cognitive Dysfunction, Doença de Huntington, Agnosia, Disfunção Cognitiva

## Abstract

Huntington's disease (HD) is an inherited disease that leads to an inexorable progression of motor, cognitive and psychiatric disturbances. In the initial stages, the symptoms are not clearly disabling, and the patient may present a lack of awareness about the symptoms themselves, which we call anosognosia. However, anosognosia might not justify all passivity of the HD patient in face of the diagnosis. Patients may also experience the denial of illness, as a stage of grief, expected to happen in the face of the diagnosis of any neurodegenerative disorder. In addition, people with HD tend to be more apathetic, and more silent, in regular consultations. In the present article, the authors express a point of view, discussing the behavior of the HD patient, in which there is a multifactorial passivity, in the face of the diagnosis and of the disease itself. Having the proper knowledge of this situation may prepare the neurologist to better understand the patient and the evolution of the disease.

## Resumo

A doença de Huntington (DH) é uma doença hereditária que leva a uma progressão inexorável de distúrbios motores, cognitivos e psiquiátricos. Nos estágios iniciais, os sintomas não são claramente incapacitantes e há uma falta de consciência sobre os próprios sintomas, o que chamamos de anosognosia. No entanto, anosognosia pode não justificar toda a passividade do paciente de HD diante do diagnóstico. Os pacientes também podem vivenciar a negação da doença, como um estágio de luto, o que é esperado acontecer diante do diagnóstico de qualquer doença neurodegenerativa. Além disso, as pessoas com DH tendem a ficar mais apáticas, mais silenciosas, nas consultas regulares. No presente artigo, os autores expressam um ponto de vista, discutindo acerca do comportamento do paciente com DH, em que há uma passividade multifatorial, frente ao diagnóstico e diante da doença em si. Ter conhecimento sobre essa situação pode preparar o neurologista para entender melhor o paciente e a evolução da doença.

## INTRODUCTION

Huntington's disease (HD) is an inherited disease that leads to an inexorable progression of motor, cognitive, and psychiatric disturbances. 1 Along the journey of the HD patient, from the first symptom to the final diagnosis, many years and many doctors shall pass. But when a patient walks through the door, what about him can you observe? The patient sits in front of you and says: “I don't have anything.” And assuming he believes it to be true, does not that say something? And how much a patient with a movement disorder can tell us, just at the glance of a slight wiggle, without saying a single word? How often will a patient with HD walk into the clinic, sitting at the table, without saying anything? Well, by not saying something, it says something about the patient too, doesn't it? Without talking, without being able to get close, how much a chorea might show you? How much might a patient who moves a lot, and says little, unveil himself?

## DISCUSSION

Huntington's disease is a phenomenologically rich disease. In addition to chorea, its hallmark, many other movement disorders have been described. 1 2 3 4 Psychiatric and cognitive symptoms have also been the aim of deeper understanding since they are associated with great caregiver burden. 5 The symptoms of HD are initially subtle, 1 6 and very often, the complaint that will lead the patient to the first appointment will come from a family member or a close friend. It may happen, first, because at the initial stages the symptoms are not clearly disabling. Frequently, the HD patient seems to lose initiative and become more passive and quieter, even when interspersed with moments of agitation and aggressiveness. Moreover, there is a lack of awareness about the symptoms themselves, which we call anosognosia. 7 Anosognosia was a term coined by Babinski, 7 but it was first recognized in HD before the gene discovery, 8 and it is now a well-documented feature of HD 8 9 10 11 12 and other degenerative diseases. 13 14 15 16 People with HD may show reduced awareness of mental and physical changes in themselves, underestimating the presence or the severity of involuntary movements, not perceiving behavioral changes and cognitive difficulties, and, consequently, underreporting these symptoms to the family and to the doctors. The patient tends to not use medication properly, attend clinics, or report difficulties in daily routine, leading to a dangerous overestimation of their abilities, generating a huge caregiver burden. 9 13 Symptoms awareness may vary significantly between individuals, but appears to be influenced by comprehension capacity and educational level prior to the disease, mood, and the global cognitive decline, specifically executive function. 14 15 16 Isaacs et al. found that anosognosia was present in one-third of the patients. 9 Before that, McCusker et al. 10 analyzed the PREDICT-HD data and identified that ∼50% of patients were unaware of motor features at the diagnosis. 10 11 Unawareness of executive dysfunction has been also documented in the premanifest patients in PREDICT-HD. 11 12 Surely, since cognitive function decline is inevitable in HD, it tends to cover all patients, throughout the course of the disease.

Altered awareness of motor, cognitive, and neuropsychiatric symptoms is not exclusive to HD, and is also present in Parkinson disease (PD) and other neurodegenerative disorders. 16 17 18 Interestingly, people with PD who have intact cognition, may over-report cognitive symptoms, whereas those with cognitive impairment may under-report these symptoms. 16 When comparing awareness of motor symptoms and activities of daily living impairment in HD and PD, Sitek et al. found that self-awareness of movement disorders was severely more affected in HD than in PD patients, despite comparable cognitive status, possibly supporting that anosognosia, as an organic disorder, may be predominantly associated with orbitofrontal–limbic pathology in HD. 18

However, anosognosia may not justify all the passivity of the HD patient. When the patient says: “I don't have Huntington's disease,” we have a perception that, in addition to the unawareness of self-symptoms, patients with HD may also experience the denial of illness, possibly, as a stage of grief, expected to happen in the face of the diagnosis of any neurodegenerative disorder. The five stages of grief, namely: denial, anger, bargaining, depression, and acceptance, were developed by Elisabeth Kübler-Ross, first to represent an expected variety of feelings experienced by a person who lost a loved one. 19 But the stages of grief may also explain the emotional experiences of a patient – and their family – in the face of a diagnosis of a severe or fatal disease. Deckel et al. described a study in which they investigated the neurologically based “denial of illness.” 20 The design of the study, however, may present more evidence about the HD anosognosia itself, showing that may be even more difficult to distinguish denial from anosognosia. Besides, the acceptance of a disease itself also depends on the degree of understanding of it. No one is capable of accepting something they do not understand.

Furthermore, people with HD tend to become more apathetic, and more silent, in regular consultations. Apathy is the most prevalent behavioral symptom in HD, occurring in ∼ 70% of the patients, and is correlated with the duration of the disease. 21 Also, anhedonia and abulia, defined as a lack of will or motivation, representing a possible *continuum* of the emotional deficit present in HD, may lead to a severe impact on quality of life, and, in severe stages, may lead to akinetic mutism. 21 The real limit, however, of the extent to which this passivity – or permissiveness – between “denying, not understanding, and being apathetic” is a difficult task for the attending doctor. But, perhaps, all these factors together may contribute to diminished patient self-reporting of HD symptoms, and consequently may lead to poor medication management and a challenging therapeutic approach.

The boundaries between acceptance and understanding HD are thin, but its recognition by clinicians of deficient self-awareness is crucial because of its implications for diagnosis and optimal clinical management of HD. During a normal day of work, any doctor may face questions such as: “it can't be anything else? Are you sure about the diagnosis? Can the exam be wrong?” These questions are commonly expected as a manifestation of a stage of denial. Interestingly, these questions come also from family members and companions when talking about HD. This leads to the belief that, surely, denial may be present in patients with HD. However, the lack of understanding and initiative, and the characteristic apathy of the patient, may be important factors, especially as the disease progresses.

To differentiate anosognosia from denial of illness, there are, perhaps, some interesting points to highlight. First, denial tends to be greater in the face of the initial diagnosis, especially when it is the first doctor to communicate the disease. Denial is commonly accompanied by denial from other family members. And when an issue is raised, the patient may react with emotions of anger or deliberate detachment, a possible “fight or flight” posture. And it tends to be more relevant, the greater the cognitive capacity at the time. Anosognosia, on the contrary, seems to be correlated with cognitive function decline, and gets worse as the disease progresses. Also, the patient tends to receive someone's perception of his symptoms with surprise, and maybe some disinterest, but not with anger. Moreover, anosognosia does not correlate with family members' complaints. In general, the patient underestimates the symptoms, while family members and caregivers may overreport the same symptoms.

Finally, there is a panfamilial impact in HD. Due to the hereditary nature of the disease, commonly, several family members are affected at the same time, and there is also an economic impact, since the affected family members stop contributing to the household expenses, leading to the impoverishment of the family over generations, greatly burdening healthy family members. Then, we may face the importance of the doctor on this topic. Comprehensive health care may encompass guiding and treating not just the patient, but also his family and caregivers. The doctor-patient relationship is central to providing the best support, both at diagnosis and during treatment. The doctor must assess the level of education of the patient and family members, bring the family closer in the consultation, and balance the autonomy of the patients, with their cognitive limitations and capacities still preserved. The doctor may approach the diagnosis, treatment options, evolution, and prognosis. And, when there is a clear limitation in acceptance or understanding, the professional may optimize the doctor-patient relationship, having a “damage control” approach, giving only the information that is possible to be understood, avoiding excess or multiple instructions in the consultation, and directing the treatment more emphatically, to not confuse the affected ones, and bringing more empathy. It is crucial that palliative care, a multidisciplinary approach, and also legal rights may be discussed as soon as possible.

Therefore, health professionals must be aware that anosognosia, but also the denial of the disease, directly influences the treatment of HD, and many other neurodegenerative disorders. They may occur simultaneously and many times we will not be able to distinguish each other. Nevertheless, at the boundaries between acceptance and understanding, it just may not exist a barrier between the doctor and the patient.
